# Flexible voices: Identity perception from variable vocal signals

**DOI:** 10.3758/s13423-018-1497-7

**Published:** 2018-06-25

**Authors:** Nadine Lavan, A. Mike Burton, Sophie K. Scott, Carolyn McGettigan

**Affiliations:** 1 Department of Psychology, Royal Holloway, University of London, Egham, Surrey, UK; 2 Department of Psychology, University of York, York, UK; 3 Institute of Cognitive Neuroscience, University College London, London, UK

**Keywords:** Voice, Identity, Variability, Familiarity

## Abstract

Human voices are extremely variable: The same person can sound very different depending on whether they are speaking, laughing, shouting or whispering. In order to successfully recognise someone from their voice, a listener needs to be able to generalize across these different vocal signals (‘telling people together’). However, in most studies of voice-identity processing to date, the substantial within-person variability has been eliminated through the use of highly controlled stimuli, thus focussing on how we tell people apart. We argue that this obscures our understanding of voice-identity processing by controlling away an essential feature of vocal stimuli that may include diagnostic information. In this paper, we propose that we need to extend the focus of voice-identity research to account for both “telling people together” as well as “telling people apart.” That is, we must account for whether, and to what extent, listeners can overcome within-person variability to obtain a stable percept of person identity from vocal cues. To do this, our theoretical and methodological frameworks need to be adjusted to explicitly include the study of within-person variability.

## Introduction

The human voice provides a multitude of cues to person identity – from only a short recording of a voice, listeners can discriminate between speakers, recognise them as familiar and identify them by their names (Kreiman & Sidtis, 2011). Variations in the anatomy of each individual’s vocal apparatus, such as the thickness of the vocal folds, differences in the shape of a person’s palate, and the dynamic use of the vocal tract, give rise to differences in pronunciation, accent and other idiosyncratically marked features of a person’s vocal inventory (see Scott & McGettigan, 2015, for a review). The acoustic consequences of these differences between individuals allow listeners to process a speaker’s identity from their vocal signals only.

Voice recognition is a remarkable feat: Human vocal behavior is in fact highly variable and complex. For example, the act of speaking requires precise temporal and spatial coordination of a number of anatomic structures including the lips, jaw, tongue, and the vocal folds within the larynx (or “voice box”) to produce rich and dynamic linguistic signals. Further, the flexibility of human voice production allows us to smoothly combine speech sounds into novel words, and words into unique utterances, as well as to cope with a variety of communicative pressures, such as talking to different audiences (e.g., a pet, a police officer, a language learner) and talking in different contexts (e.g., in a noisy café vs. in a quiet library). Taken together, these properties of the vocal system introduce considerable within-person variability in the human voice, such that its physical properties are highly dependent on the content and context of the behavior being performed. To some extent, listeners can overcome this variability to extract a relatively stable percept of an individual. Yet, there are striking examples of when this ability fails, as the field of earwitness research can readily attest (Read & Craik, 1995; Saslove & Yarmey, 1980).

Broadly described, identity perception depends on two fundamental processes: telling different people apart, while also maintaining constancy in the perception of individuals across different and varying instances (i.e., “telling people together”, Burton, 2013). Surprisingly, however, the literature on voice perception has mainly focused on characterizing the former – telling people apart using stimuli and paradigms that prioritise perception of the distinguishing features of individual voices. Where the speech perception literature has emphasized the need to account for within-person variability (alongside between-person variability) in achieving perceptual constancy (Johnson, 2005; Liberman & Mattingly, 1985; Massaro & Chen, 2008; Pisoni, 1997; Weatherholtz & Jaeger, 2015), the voice-identity literature has not: The standard approach to studying vocal identity perception has been to explicitly control away *within*-person variability, for example by representing voices through selecting exemplars of controlled sentences, words, or even vowels, typically produced in a uniformly neutral tone of voice, and obtained from a single recording session. We will explain how this approach overlooks some crucial aspects of how the human voice works.

The face perception literature has already highlighted the need to study within-person variability: within-person variability in faces is substantial and the challenges it poses for face identity perception have been described in a number of studies (e.g., Burton, 2013; Jenkins, White, Montfort, & Burton, 2011). Intriguingly, studies have also highlighted that within-person variability may not always pose challenges to viewers but may indeed be essential for learning new identities (Burton, Kramer, Ritchie & Jenkins, 2016; Murphy, Ipser, Gaigg & Cook, 2015; Ritchie & Burton, 2017). The study of within-person variability in faces has thus opened up many fruitful new avenues of investigation, progressing the field.

Here, we argue that – as was the case in the face perception literature (Burton, 2013) – the historical focus on telling people apart has restricted our understanding of voice-identity perception to a limited set of conditions. This not only limits our understanding of the robustness of the perceptual system to within-person variability, but it may also obscure some of the information critical to the formation of long-term perceptual representations of speaker identity (Burton et al., 2016). If within-person variability provides the listener with diagnostic information about identity – rather than simply adding noise to the signal – then experimental methods that eliminate it can never fully address the problem of identity perception and learning. In short: half of the field of voice-identity perception is missing. This paper documents why this is an important omission, and proposes future directions for the field to redress the balance.

In the sections that follow, we describe sources of this variability in the voice, followed by a review of studies that have explored the effects of within-person variability on identity perception. We then synthesise these emerging data on within-person vocal variability with complementary findings from the face perception literature. Finally, we present key questions for future research into identity processing from vocal signals.

## Sources of within-person variability in the voice

**Fig. 1:**
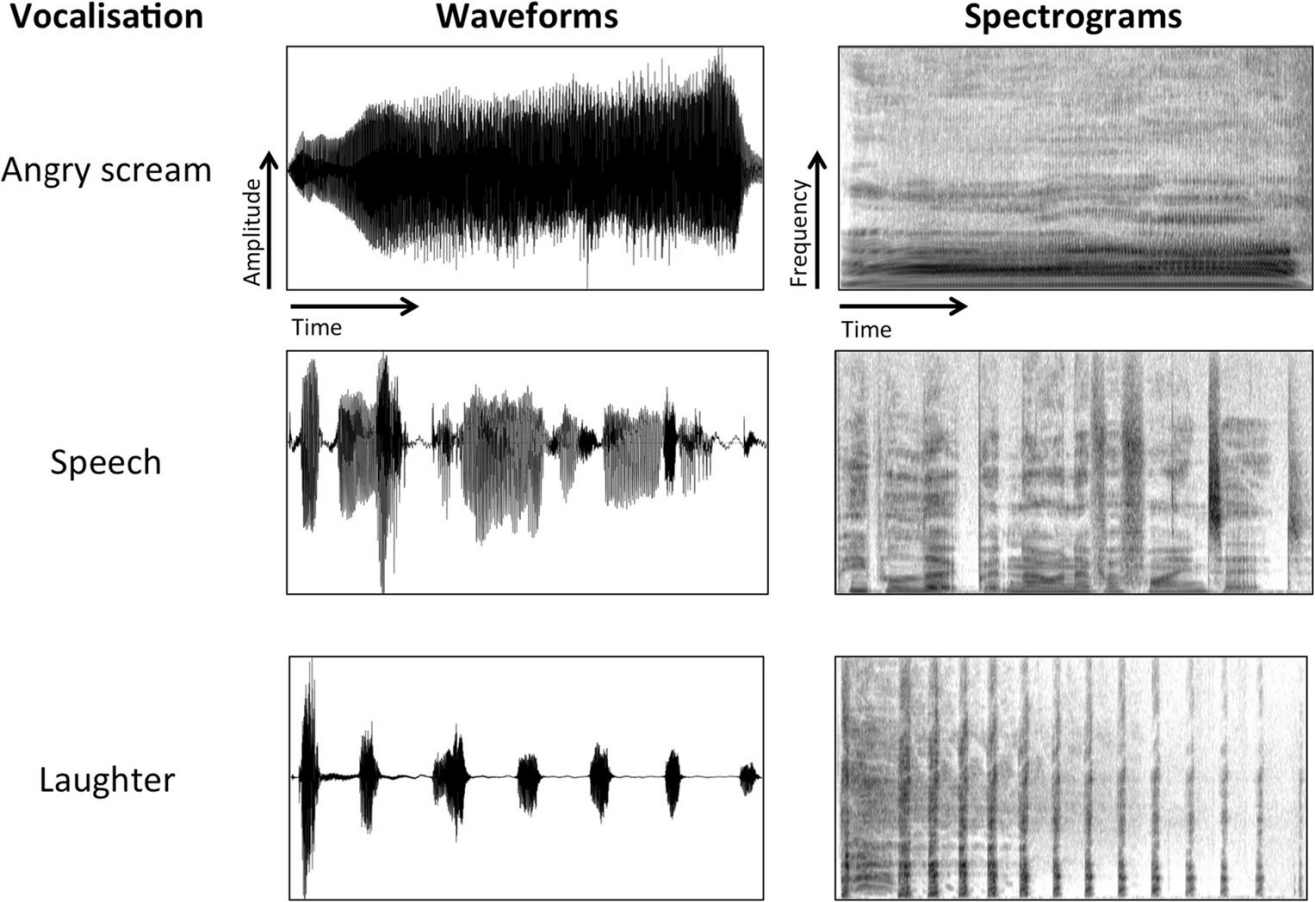
Waveforms and spectrograms of three different vocalizations illustrating the variable physical features of human vocalizations

Within-person variability in our vocal signals is substantial: we volitionally modulate our voices to express our thoughts and intentions or adjust our vocal outputs to suit a particular audience, speaking environment, or situation. The sound of our voices and the type of vocalizations we produce are additionally affected by processes over which we have little to no volitional control, such as changes in emotional states or our state of health (see Table 1). Figure 1 further illustrates how different the physical makeup of vocalizations can be: despite being of similar duration, it is apparent that all three depicted vocalizations differ substantially from each other in their physical structure. Differences in the number of onsets, type and rate of modulation of the signal and overall spectral properties of the different segments can be seen from the waveforms (left) and spectrograms (right) of the sounds.

**Table 1:** Overview of some of the volitional and spontaneous sources of within-person variability in voices

Volitional modulations	Examples
Situation-dependent modulations in clear speech	Conversational speech Reading aloud Giving a formal presentation Convincing another person of your argument Whispering confidential information
Environmental effects	Speaking over different types of background noise Speaking in a hushed voice in quiet environments
Audience-dependent modulations	Child-directed speech Pet-directed speech Speech directed at hearing-impaired individuals Speech directed at language learners
Voice artistry	Impersonation Voice acting Singing Rapping Beatboxing
Imitation and disguise	Voice imitation in indirect speech Voice disguise (forensic) through the use of e.g., accents or changes in speaking style
Spontaneous modulations	Examples
Changes across the lifespan	Developmental changes (e.g., during puberty) Age-related changed to vocal physiology and speaking style
Changes linked to mental and physical health	Speaking while having a cold (Occupational) vocal fatigue and voice loss Voice changes due to long-term habits, e.g., smoking Voice changes in depressed individuals
Changes as a result of emotional states	Non-verbal emotional vocalizations (laughter, crying) Emotionally-inflected speech

In a recent study, Kreiman, Park, Keating and Alwan (2015) attempted to empirically quantify within-person variability sampled in everyday speaking situations collected across different recording sessions – the samples included unscripted neutral, positively-valenced, and negatively-valenced speech, as well as pet-directed speech. To quantify variability, the authors extracted a number of acoustic measures, such as mean F0, formant measures, and cepstral peak prominence (CPP – indexing periodicity) and calculated the average Euclidean distance within and between talkers. They reported that within-person variability in this set of speech vocalizations was indeed large, but did not exceed the between-speaker variability in their study. Notably, how this particular sample of laboratory-induced variability relates to within-person variability in naturalistic, everyday settings remains unclear, and additional studies are needed to fully describe the extent of natural within-person variability in the voice. The section below will provide an overview of some of the sources of volitional and spontaneous voice changes.

### Volitional voice modulation: speaking styles, effects of environmental and social context

Humans dynamically adapt the way they speak depending on their audience and their physical environment in order to maximise intelligibility (see Communication Accommodation Theory; Giles, 2008). These adaptations differ from situation to situation: child-directed speech as well as pet-directed speech both involve elevated fundamental frequency (F0; correlated with perceived pitch), and what researchers have termed “exaggerated affective qualities,” compared to adult-directed speech (Hirsh-Pasek & Treiman, 1982). However, only child-directed speech features overarticulated vowels (Burnham, Kitamura, & Vollmer-Conna, 2002), suggesting that talkers accommodate flexibly to the needs of the audience – babies must learn to talk, therefore they need greater support in distinguishing key phonemic contrasts in their language. Similar changes, with regard to overarticulation of specific speech segments, have been reported for speech directed at elderly listeners (Kemper, Finter-Urczyk, Ferrell, Harden, & Billington, 1998) and in the context of speech directed at non-native speakers (Kangatharan, Uther, & Gobet, 2012; but see Hazan, Uther, & Granlund, 2015). Environmental factors can also affect our voices: In the presence of loud competing noises, or when required to talk across a distance, speakers will increase their vocal effort, which has been linked to higher intensity and F0 as well as longer vowel durations (Summers, Pisoni, Bernacki, Pedlow, & Stokes, 1988; Traunmüller & Eriksson, 2000).^1^ Conversely, when in a quiet environment, speakers may start to whisper or use hushed speech, which is marked by the reduction or complete removal of voicing (Ito, Takeda, & Itakura, 2005; Traunmüller & Eriksson, 2000).

Even in situations lacking specific pressures to enhance speech clarity, speakers frequently adopt modified styles of speaking: for example, conversational speech is distinct from read speech, with differences occurring in the speech rate, overall F0 and range of F0 (Hazan & Baker, 2010). Similarly, theatrical speech produced by actors differs from conversational speech in its spectral properties (Raphael & Scherer, 1987). Speakers can volitionally modulate their voice to convey social traits and attitudes (e.g., confidence, attractiveness; Hughes, Mogilski & Harrison, 2014); speech that is intended to be affiliative, dominant, or submissive differs in acoustic characteristics, such as speech rate, hesitations, and loudness (Carli, LaFleur, & Loeber, 1995). Thus, in addition to modulations of intelligibility, speakers are able to volitionally encode meaningful non-verbal information about themselves and their intentions into their voices, which listeners can in turn decode and use to make judgments about a speaker or situation.

Voice artistry offers insights into some of the most extreme examples of volitional voice change, showcasing the flexibility of human vocal signals. Impressionists are able to produce vocal signals that sound convincingly like another (familiar) person, while other voice artists regularly cross age, accent, and/or gender boundaries during their voice work: notably, the cartoon character Bart Simpson, a 10-year-old boy, has been voiced by female voice artist Nancy Cartwright (who was born in 1957; Cartwright, 2000) since the late 1980s. Human beatboxing goes a step further, in that performers produce sounds with their voices that perceptually do not even resemble human vocalizations but are more like musical instruments or electronically manipulated sounds (Proctor et al., 2013; Stowell & Plumbley, 2008). Voice imitations are, however, not limited to expert groups: In everyday communication, speakers will volitionally imitate or produce a caricatured version of another person’s voice to mark reported speech in conversation: changes in F0, F0 variability, speech rate, overall timing, and phonation type (e.g., breathy voice, creaky voice) have been observed in such contexts (Klewitz & Couper-Kuhlen, 1999; Jansen, Gregory, & Brenier, 2001). The nature and extent of those changes will depend on the particular characteristics of the target speaker that the imitator wants to convey. Another striking example of volitional voice change is voice disguise: voices can be disguised in any number of ways, through accent imitation, changes in speaking styles, and changes in F0 or phonation style (Eriksson, Llamas, & Watt, 2010). Finally, the singing voice has been shown to differ from the speaking voice in a number of features, for example, in its resonant properties (i.e., formant structure) and the presence of vibrato (Sundberg, 1977; Sundberg 1995; Horii, 1989). When singing, a person’s natural speech rhythm and intonation are additionally adapted to align with a predefined musical rhythm and melody.

### Non-volitional modulations: changes across the lifespan, emotions, and physical and mental health

Our voices change in ways over which we have little to no volitional control. Such changes occur on different timescales: while emotional experiences can affect the sound of a voice from moment to moment, our voices will change over the course of our lives as a result of developmental changes and aging. Children’s voices, for example, undergo significant changes during puberty, when the size and thickness of their vocal folds increases and thus lowers the F0 of their voices (Decoster & Debruyne, 1996; Hazan, 2017). These changes are especially pronounced in males, leading to the characteristic sexual dimorphism of vocal pitch in humans (e.g., Puts, Gaulin, & Verdolini, 2006). Further changes occur in the voices of elderly people: the F0 changes (typically decreasing in female voices and increasing in males), speech rate gets slower, and there are indications that the overall quality of a voice is affected by the aging of the vocal folds (Linville, 1996). How quickly and dramatically a voice changes will depend on general health and fitness (Linville, 1996), as well as long-term habitual behaviors such as smoking (Damborenea et al. 1998; Sorensen & Horii, 1982) or a person’s occupation (Russell, Oates, & Greenwood, 1998; Smith, Gray, Dove, Kirchner, & Heras, 1997): Chronic vocal fatigue, voice loss, and other voice problems are, for example, frequently reported for teachers, singers, and other occupations requiring intense voice use (Williams, 2003).

Spontaneous modulations of the voice have been reported in emotional speech and non-verbal emotional vocalizations – these can occur rapidly, in the order of milliseconds to seconds. Drastic modulations of F0 have been linked to changes in emotional arousal, where F0 increases with increasing emotional arousal (see, e.g., for laughter, Bachorowski, Smoski, & Owren, 2001, Bachorowski & Owren, 2001, Lavan, Scott, & McGettigan, 2016a; Vettin & Todt, 2004). Further emotion-specific acoustic signatures have been reported, affecting the temporal and spectral properties of vocalizations in addition to changes in F0 (Juslin & Laukka, 2003; Sauter, Eisner, Calder, & Scott, 2010). Additionally, other relatively short-term changes in the sound of a voice can be induced by stress (Tolkmitt & Scherer, 1986), sleep deprivation (Harrison & Horne, 1997; Vogel, Fletcher, Snyder, Fredrickson, & Maruff, 2011) and the state of the physical or mental health of a person (Cannizzaro, Harel, Reilly, Chappell, & Snyder, 2004; Pribuisiene, Uloza, Kupcinskas, & Jonaitis, 2006).

These are only some of the examples of the ways the voice of a single person can vary. The acoustic sources of within-person variability in the voice differ qualitatively from each other: while some features are *modulated* by changes in vocal behaviour (e.g., F0 in pet-directed speech), other features may be *absent* under certain conditions (e.g., a lack of F0 for whispered speech). Considerations of varying timescale and volitional control further complicate the picture (changes across the lifespan vs. short-term changes). These modulations are not incidental noise; they emerge from intrinsic physiologic properties of the human voice and thus must be modeled as such. That is, the nature and scale of within-person vocal variability renders insufficient any model of vocal identity that represents the individual as a point within an acoustic space – yet such an approach, prioritizing between- over within-person cues, has provided the conceptual backdrop to the vast majority of existing literature on voice identity.

## Studies of voice-identity perception addressing within-person variability

While many studies of voice-identity processing to date have probed listener’s aptitude to recognise, discriminate, and learn vocal identities while minimizing within-person variability, a relatively small number of studies has attempted to explicitly characterise how within-person variability affects the extraction of person identity from the voice. In these studies, within-person variability is introduced into sets of vocal signals to address questions of how identity perception is affected by the amount and type of information available, and to probe the extent to which listeners can generalize identity perception across variable signals. The prevailing finding from such work is that variability in the voice has detrimental effects on speaker perception, especially when listeners are unfamiliar with the speakers. An illustration of this can be found in a voice-sorting study (Lavan, Burston, & Garrido, 2018): using an identity sorting paradigm pioneered in the face perception literature (e.g., Andrews et al., 2015; Jenkins et al., 2011; Redfern & Benton, 2017; Zhou & Mondloch, 2016; see below), unfamiliar listeners sorted 30 brief audio clips (~3 s in duration) into perceived identities. The audio clips were extracted from a popular TV show to include natural vocal variability. Listeners, who reported to never have seen this show and were thus unfamiliar with the voices, perceived a larger number of identities than was present in the stimulus set (four to nine identities perceived; veridical number: two identities). Errors were mainly restricted to *telling people together*, while fewer errors occurred for *telling people apart*: that is, unfamiliar listeners struggled to generalize identity information across variable signals, often labeling variable sounds from the same voice as belonging to different perceived identities. This closely mirrors previous findings from the face perception literature (see below).

While listeners in this voice-sorting study were presented with vocal signals including natural, uncontrolled within-person variability, other studies have explicitly manipulated specific aspects of voice production to enhance within-person variability in a controlled way. Using speaker-discrimination paradigms, studies generally show that performance drops when listeners are asked to make judgments across such variable vocal signals. Reich and Duke (1979) conducted a study in which listeners were asked to discriminate unfamiliar speakers from pairs of sentences. For each pair, one sentence was produced in an undisguised voice and the other was either undisguised or disguised (e.g., hoarse voice, extremely slow speech, and hyper-nasal speech). Speaker discrimination performance was significantly worse for pairs that included both disguised and undisguised speech (and thus more vocal variability) compared with pairs of undisguised sentences. Similarly, speaker-discrimination accuracy was reduced when listeners were asked to match identities from spoken words to sung words (Peynircioğlu, Rabinovitz, & Repice, 2017) or across different types of vocalizations (e.g., a spontaneous laugh vs. a series of vowels). In the latter case, performance in some conditions was no different to chance (Lavan, Scott, & McGettigan, 2016b). Wester (2012) showed that speaker discrimination across different languages is more difficult compared to discriminations within the same language. For studies of earwitness accuracy, it was also shown that listeners’ ability to identify a voice from a line-up decreased dramatically when vocal variability due to emotional content was introduced between study and test (Read & Craik, 1995; Saslove & Yarmey, 1980). In these speaker-discrimination studies, within-person variability therefore consistently affects listeners’ performance: when unfamiliar with a voice, listeners can only generalize to a limited extent across differences between two vocal signals to accurately extract a stable percept of speaker identity. With no robust perceptual representation of the voice (also referred to as familiar voice patterns; Kreiman & Sidtis, 2011; Sidtis & Kreiman, 2012) being available for unfamiliar voices, within-person variability is therefore likely to be mistaken for between-person variability (see Lavan et al., 2018).

### How to ameliorate the effects of within-person variability: the case of familiarity

Within-person variability seems to reliably disrupt identity processing for unfamiliar voices. Familiarity with a speaker has, however, been shown to partially ameliorate these effects. In the voice-identity-sorting task (Lavan et al., 2018) described above, a group of listeners familiar with the voices completed the study alongside listeners who did not know the voices. These familiar listeners perceived only three to four identities (compared with the four to nine identities perceived by unfamiliar listeners). Thus, despite making some errors, familiar listeners came to solutions that were closer to the veridical number of two identities. Similarly, in their study looking at speaker discrimination across different vocalizations, Lavan et al. (2016b) found that a group of psychology students who had been lectured by the speakers whose voices were included in the study performed better at speaker discrimination across all conditions, compared to the listeners who were unfamiliar with the voices. Nonetheless, these familiar listeners in the Lavan et al. study (2016b) showed far from perfect performance. This is perhaps not surprising, given that this group had no regular prior experience with the particular vocalizations used (laughter, trains of vowels), but it gives us some insights into the limits of familiarity advantages: it appears that without explicit experience with a particular type of vocalization, extensive within-person variability can still present significant challenges in mapping examples of that vocalization onto the already learned representations.

This finding is partially confirmed by studies of voice-identity perception using manipulations of language familiarity instead of speaker familiarity. While explicit familiarity with a speaker or a voice has been shown to afford advantages for voice-identity processing, familiarity with other properties of the stimuli can also aid identity perception. For example, it is easier to discriminate and recognise speakers in one’s own language (e.g., Perrachione, Del Tufo, & Gabrieli, 2011; Perrachione, Pierrehumbert, & Wong, 2009; Winters, Levi, & Pisoni, 2008). It has been argued that this effect stems specifically from familiarity with the phonology of a language, which allows listeners to access and/or perceive cues to speaker identity. Evidence in favor of this claim has been provided by a study of Perrachione et al. (2011): individuals with dyslexia were tested on a speaker recognition task and compared to a control group. Dyslexia is a learning difficulty that has been linked to impoverished phonological processing, which is thought to disrupt access to linguistic representations via speech (and reading). When judging identity from stimuli produced in an unfamiliar language, the absence of familiar phonological information in the stimuli led to similar task performance for both listener groups. However, when judging identity from speech in their native language, the control group showed an advantage in line with their presumed superior access to familiar phonological cues. Further evidence of phonological familiarity effects can be found in a study by Zarate et al. (2015). In a speaker-discrimination task across different languages, the authors tried to pick apart the influences of different types of linguistic information and their accessibility on speaker recognition accuracy. The presence of phonological information per se (i.e., spoken sentences in Mandarin, German, English, or “Pseudo-English”) generated more accurate speaker recognition than non-verbal vocalizations (i.e., laughter, crying, coughs, grunts). Further, there was a significant advantage for the native language (English), regardless of whether this was semantically intelligible (i.e., no difference between English and Pseudo-English). Intriguingly, familiar language advantages are even apparent for (unintelligible) time-reversed speech (Fleming, Giordano, Caldara, & Belin, 2014, though see Skuk & Schweinberger, 2014) and when listeners have only been passively exposed to the language, without understanding it (Orena, Theodore, & Polka, 2015).

Overall, familiarity with a speaker or a stimulus provides listeners with an advantage in identity processing. In contrast to unfamiliar listeners, familiar listeners appear to be able to better generalize the information from familiar signals (e.g., speech in a familiar language) to less familiar signals (e.g., laughter or speech in another language). This may reflect differences in accessibility of information due to familiarity and/or the ability to match vocal signals to a specific representation, although it appears that passive exposure to relevant stimulus properties can be sufficient to afford some advantages. How well listeners perform may depend on how different the vocal signals in question are from each other (in the case of discrimination tasks) or how much a given signal differs from an existing mental representation of a voice (in a recognition task). Relatedly, success in generalization may depend on how stable and comprehensive a representation of the voice (and vocalization) a listener has formed. Despite familiarity, identity processing can still fail under certain circumstances: Wagner and Köster (1999) report that listeners were close to chance performance when asked to recognise familiar individuals speaking in a falsetto voice, despite being exposed to relatively long samples of speech (15 s in duration). The robustness and stability of a representation are likely linked to the type of familiarity with a voice (e.g., personally/intimately familiar vs. famous voices, see Kreiman & Sidtis, 2011), the type and duration of exposure (e.g., passive exposure vs. active interactions; variable settings vs. fairly uniform settings) and other factors (e.g., whether we like/dislike a person). Future research is required to further explore how these factors interact with the processing of variable vocal signals.

### Differences in vocalization types, differences in performance

The studies above probed the question whether, and to what extent, listeners can make identity judgments across variable vocalizations. Another set of studies has looked at how well listeners can extract identity information from vocalizations that diverge from what might be considered normal, neutral speech. In such vocalizations, acoustic cues to identity may be drastically modulated or may even be absent (see above). Studies comparing identity perception from whispered speech and voiced (normal) speech report worse performance for speaker discrimination and recognition from whispered sounds (Abberton & Fourcin, 1978; Bartle & Dellwo, 2015; Orchard & Yarmey, 1995; Pollack, Pickett & Sumby, 1954; Yarmey, Yarmey, Yarmey, & Parliament, 2001). Here, the drop in performance can be attributed to the absence of acoustic cues to voice identity: in whispered speech the F0 of a voice is missing, leaving listeners with relatively less diagnostic information regarding a speaker’s identity. However, in no case did performance drop to chance levels, suggesting that there is sufficient information in whispered speech that allows processing a speaker’s identity. Similarly, in their speaker-discrimination task, Lavan et al. (2016b) reported impaired performance for spontaneous laughter – a follow-up study suggested that it is the difference in the underlying production mechanisms that leads to impairments for spontaneous laughs (Lavan et al., 2018). This finding presents the intriguing possibility that cues assumed to be stable indicators of person identity are missing in spontaneous vocalizations – or indeed, that they are *added* during volitional vocalizations. Identity perception may thus be differentially challenging for different vocalizations.

An implication of this work is that existing accounts of person identity perception from the voice, being based primarily on intelligible, neutral speech, may be yet further underspecified: Speech, especially when produced in a language familiar to the listener (see Goggin, Thompson, Strube, & Simental, 1991; Orena et al., 2015; Winters et al., 2008), is uniquely rich in accessible cues to speaker characteristics and identity, including regional accent, lexical content, and individual differences in pronunciation. Such speech-specific cues have been shown to be of great importance for extraction of speaker characteristics and identity (e.g., Remez, Fellowes, & Rubin, 1997; Zarate et al., 2015) but are largely absent in, for example, non-verbal vocalizations such as laughter or even isolated vowels. Furthermore, specific acoustic cues thought to be important for identity perception, such as information about a speaker’s F0, are well preserved in natural speech but notably absent or distorted in other contexts (e.g., whispering, laughter). Thus, using fully voiced, emotionally neutral speech samples may have provided relatively favorable conditions for identity perception in previous studies, leading to overestimations of participants’ ability to process identity from vocal signals.

Overall, within-person variability has been shown to have striking effects on identity perception from vocal signals, often presenting significant challenges for listeners. While familiarity can partially ameliorate these detrimental effects, unfamiliar listeners appear to consistently misperceive within-person variability as between-person variability. Additionally, in certain vocal signals crucial information may not be encoded (e.g., whispered speech, spontaneous vocalizations) or may not be readily accessible to listeners (e.g., speech produced in an entirely unknown language, Perrachione, et al., 2011; Perrachione, et al., 2009; Winters, et al., 2008). We therefore argue that the study of within-person variability not only opens up exciting new avenues of research, but that it is, in fact, essential to account for the varied effects of within-person variability in theoretical and methodological frameworks applied to voice-identity perception.

## Identity perception and within-person variability: perspectives from face perception

**Fig. 2:**
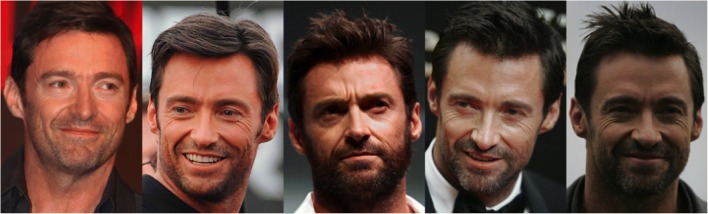
Multiple photos of the same actor. There are very large differences between these images, but viewers familiar with the actor have no problem recognizing him in each of them. Image attributions from left to right: Eva Rinaldi (Own work) [CC BY-SA 2.0], Grant Brummett (Own work) [CC BY-SA 3.0], Gage Skidmore (Own work) [CC BY-SA 3.0], Eva Rinaldi (Own work) [CC BY-SA 2.0], Eva Rinaldi (Own work) [CC BY-SA 2.0]

A similar line of research has also been gaining popularity over the last few years in the field of human face perception (Burton, 2013; Burton et al., 2016; see Fig. 2). While researchers have for some time drawn parallels between face and voice processing (Campanella & Belin, 2007; Yovel & Belin, 2013), this more recent line of research on within-person variability in identity perception allows us to uncover striking similarities between face and voice perception from an entirely novel perspective.

As with voice studies, experiments probing the perception of facial identity have in the past tended to focus on between-person variability, using highly controlled stimuli that minimize variability in the depiction of any given individual – for example, using head-on photographs of faces, taken in a single session and with controlled lighting conditions. Outside of laboratory conditions, images of faces vary naturally in, for example, viewpoint, expression, lighting and hairstyle; quantitative analyses of variability across multiple photographs of the same people indicate that within-person variability is substantial (see Fig. 2; Burton et al., 2016). While the processing of familiar faces appears to be largely unaffected by this within-person variability, identity perception from unfamiliar faces becomes highly unreliable in the context of variability (Jenkins et al., 2011; for a review, see Young & Burton, 2017). The voice perception literature has followed a similar path: despite dealing with inherently variable signals, studies of voice-identity perception have frequently eliminated variations within individual voices (both within and across vocalizations) to focus on the processing of between-person cues in restricted vocal contexts (i.e., neutral speech). As with perception of unfamiliar faces, within-person variability has been shown to have detrimental effects on the extraction of identity from unfamiliar voices. but familiarity can ameliorate some of these adverse effects (see above).

### Within-person variability as an informative signal

A recent finding from the face perception literature has opened up further intriguing avenues of research suggesting that within-person variations in facial appearance can *benefit* the viewer in certain contexts, by providing useful person-specific information. Burton et al. (2016) report an analysis showing that within-person variability across many images of a face is highly person-specific (see Fig. 2). While no similar analyses exist for voices, Burton et al.’s (2016) finding resonates with the fact that studies have struggled to establish a fixed set of acoustic cues that encode identity across all voices (Kreiman & Sidtis, 2011) – that is, the acoustic parameters that separate individual voices in one group of speakers may lack generalizability because they have been determined using undersampled person-specific information. Interpreting their observations from faces, Burton et al. (2016) argued that person-specific variability itself may act as a signal and may thus be crucial for identity perception and identity learning (Burton et al., 2016,2). If voices behave like faces, it may be the that voices vary in person-specific ways and this variation may thus allow for the formation of unique patterns or representations for a specific voice; the perceptual salience and weighting of the different acoustic cues is also likely to depend on each individual listener and their previous experience with similar stimuli (see discussion of language effects).Face perception researchers have recently begun to test the proposal that variability may be essential to learning. To date, studies have shown evidence for improved learning of facial identity when viewers were exposed to highly variable sets of stimuli during training (Murphy et al., 2015; Ritchie & Burton, 2017). However, Kramer et al. (2016), noted that training images must lie within the range of *natural variability*, as participants were unable to successfully learn identities from TV shows in which the image contrast was reversed or the picture was presented upside down.

Only little work is available to directly show advantages for variability in voice-identity perception, but there is evidence that, for example, increasing the length of a voice sample (and thus the number of individual phonemes contained in it) leads to more accurate voice-identity processing (Schweinberger, Herholz, & Sommer, 1997), over and above the effects of increasing the acoustic duration (Bricker & Pruzansky, 1966). Bricker and Pruzansky (1966) argue that listeners process voice identity by mapping out a speaker’s phonemic inventory, and that this process is enriched by increasing linguistic variability. This is, however, only one specific type of within-person variability, linked to the number of meaningful and familiar linguistic units in speech. It remains to be established whether, for example, emotional states or different speaking styles would behave in similar ways. It furthermore remains to be formally established whether exposure to high-variability stimuli would aid the learning of a vocal identity. Whether high variability exposure is useful during voice-identity learning may also depend on the kinds of variability to which a listener is exposed; research on language effects has shown that speaker cues must be accessible to the listener in order to be informative. Future research should therefore aim to determine which aspects of within-person variability represent informative signals, and which must be treated as noise (see Kramer et al.’s [2016] notion of natural variability).

### Face and voice processing: modality-specific versus amodal processes

The literatures on face and voice-identity perception have to some degree evolved in parallel. Consequently, many concepts and experimental paradigms that were initially pioneered in the face-perception literature have later been adapted for the auditory modality, and many similarities between face and voice processing have been described (Yovel & Belin, 2013). Based on these observations, it has been proposed that the processing of faces and voices might indeed operate in equivalent ways, culminating in amodal stages supporting the formation and processing of complex, biologically-relevant categories (Campanella & Belin, 2007).

Previous research in the field has provided us with invaluable insights into the cognitive processes involved in voice-identity processing. It needs to be kept in mind, however, that faces and voices differ fundamentally from each other as physical signals. The most obvious difference is that faces are mainly studied using visual stimuli, while voices are auditory stimuli. Due to the differences in their physical properties, direct comparisons of features across these two modalities are not possible: there is no fundamental frequency in faces, and visual contrast does not affect the sound of a voice. Thus, salient features for identity processing and sources of variability indeed appear to be entirely modality-specific. Additionally, voices are inherently dynamic: A voice does not exist without an action, and thus by default information must be encoded over time. In contrast, faces can exist even without an action taking place – while emotional states or idiosyncratic expressions are encoded in the movements of a face, a static picture is in many cases enough to decode identity, emotional state, and many other characteristics (e.g., impressions of personality traits; Sutherland, Oldmeadow, Santos, Towler, Burt, & Young, 2013). While the voice perception literature has, and will continue, to greatly benefit from taking established methods and paradigms from the face-perception literature to probe novel questions in the auditory domain, it cannot be assumed that faces and voices are perceived in similar ways at all processing stages. Likewise, not all concepts and paradigms can be easily adapted across modalities. Thus, care needs to be taken to meaningfully draw parallels across modalities; further, researchers should be prepared to encounter – and account for – both similarities and differences between identity perception from faces and voices.

## Future directions for studies of voice-identity perception

Based on the body of evidence showing striking effects of within-person variability on voice (and face) identity processing, a number of key questions emerge for onward research aiming to describe voices and understand the mechanisms by which identity is perceived from vocal signals.

### Describing voices using computers: are there unique “voice-prints”?

Efforts in the mid to late 20th century aimed at creating unique “voice prints” that were visual representations derived from vocal signals, analogous to unique fingerprints. The validity of this approach was, however, largely dismissed as a result of studies showing poor matching across voice prints from variable vocal signals (e.g., disguised voices) – furthermore, at the time there were prohibitive technical limitations on performing the required large-scale acoustic analyses due to a lack of suitable computing resources (Hollien, 2002). Over the last years, however, automatic speaker recognition and verification algorithms (developed for forensic and non-forensic purposes) have reintroduced the notion of a “voice print,” using pattern recognition algorithms to identify diagnostic features in an individual’s voice. These “voice prints” represent the abstract models or templates of a speaker’s voice, and suggest that, within the constraints of a given algorithm, speaker-specific templates can indeed be derived from vocal signals. Given the large within-person variability in vocal signals, computational approaches may be able to formally establish whether within-person variability is truly person-specific and, if this is the case, describe the nature of this variability (see Burton et al., 2016 for faces). It should, however, be noted that while quantifying within-person variability in voices may be possible, it is unclear if and how such computational representations of voices relate to human identity perception: algorithms may include acoustic features in their computations that are not perceptually salient and therefore non-diagnostic to human listeners. Furthermore, it is likely that listeners flexibly weigh acoustic cues in idiosyncratic ways, making it a highly complex task to quantify truly generalizable acoustic descriptors of voice identities. Researchers of human perception may nonetheless be able to take advantage of such approaches, using them as proofs of concept to show that each voice is indeed unique (given a large enough sample). Whether and how such computational representations of voices and their variability then align with mental representations of voice identities in humans is an open question that can then be tested empirically.

### Representations of voice identities: what is their nature and how are they formed?

Many studies show that humans can learn voice identities from just a brief exposure: after listening to voices for just a few trials, listeners can accurately perform forced-choice recognition or old/new judgments on them (e.g., Aglieri, Watson, Pernet, Latinus, Garrido, & Belin, 2017; Fontaine, Love, & Latinus, 2017; Papcun, Kreiman, & Davis, 1989; Von Kriegstein & Giraud, 2006). Representations that are built up over such a short amount of time are, however, likely to be fairly unstable and may only result in reliable task performance in very specific (lab-based) contexts: the earwitness literature can otherwise readily attests to the lack of reliability for voice identifications from brief exposures (Clifford, 1980). It could therefore be argued that in order to have a comprehensive and robust representation of a person’s identity from their voice alone, a listener needs to have a wide experience of that person’s vocal repertoire, including speech in different contexts, and non-verbal vocalizations of different types (affective, such as laughter and crying, and non-affective, such as coughing). Based on varied experience with a voice, a listener might build a unified perceptual model of a person’s vocal tract, including the degrees of freedom of its articulators, and its dynamics under varying conditions. For such a model, listeners encountering a novel type, or variation, of sound from a familiar person, would be able to successfully reverse-engineer the sound via the learned vocal tract characteristics of the speaker who produced it. Alternatively, voice identification may be the result of an exemplar-based encoding of a person’s vocal behaviors in memory, such that any new instance of a vocalization is matched with the closest-fitting stored exemplar to achieve recognition (see Johnson, 2005, for similar accounts from the perspective of speaker normalization). Finally, these two hypotheses could be partially combined: while abstracted models may be formed within vocalization or speaking mode (e.g., laughter vs. speaking vs. falsetto), they may not culminate in a single unified representation but may remain vocalization-specific due to the profound differences between types of vocalizations and speaking modes (see Lavan et al., 2016b, for a discussion). Along any of these hypotheses, variable signals from a single voice could – given a certain amount of exposure – help, rather than hinder, the process of learning and familiarization. The nature of voice representations, and the mechanisms by which robust representations of a voice is achieved and maintained, must still be determined through empirical work. It further remains unclear to what extent a listener can overcome the challenges of variability – that is, can perceptual representations of highly familiar voices (e.g., partners, parents) be sufficiently specified to allow full generalization even to entirely novel vocalizations? If so, is there an upper limit on the numbers of vocal identities for which this can be achieved?

### Mechanisms of voice-identity perception: how do we go from auditory inputs to person representations?

Aside from having to determine the formation and nature of representations, we also need to understand how variable inputs are mapped to these representations. This question can be considered to be the inverse problem of how listeners achieve speaker normalization during speech perception (Johnson, 2005; Pisoni, 1997; Weatherholtz & Jaeger, 2015). Speech researchers have long been attempting to solve the “lack of invariance problem” – the observation of perceptual constancy in listening to speech despite the variability in acoustics, both within a talker and across different talkers. For example, the sound at the beginning of “sue” is acoustically quite different from the sound at the beginning of “see,” even though both instances are of the same linguistic unit /s/. Moreover, comparing the vowel in “sue” between two different talkers will reveal substantial acoustic differences, leading to partially overlapping “phoneme spaces” for different speech sounds (e.g., one person’s /u/ may be acoustically resemblant of another’s /i/). Listeners nonetheless appear to readily cope with speech signals exhibiting substantial within- and between-speaker phonetic variability, as shown by accurately recognizing spoken words. In the context of voice-identity perception, listeners are faced with similar demands: how listeners cope with variability to achieve a stable percept of identity remains unclear.

Current models of voice-identity processing provide few details on how listeners may cope with within-person variability (Campanella & Belin, 2007; Yovel & Belin, 2013; Kreiman & Sidtis, 2011; Maguinness, Roswandowitz, & Von Kriegstein, 2018). Some do, however, reference prototype-based processing as a mechanism for how voice-identity processing may work. In these accounts, incoming signals are coded as patterns of deviant features to a prototype voice – an average voice. The patterns of deviant features are then compared to a stored reference pattern of either a specific talker (in the case of familiar voices) or potentially more general templates, such as “young Glaswegian male.” While these models have been mostly applied to studying between-speaker variability (Latinus & Belin, 2011; Papcun, Kreiman, & Davis, 1989), they can be easily extended to within-person variability. In these cases, the stored reference patterns would map out the space of a single speaker’s vocalizations – in the case of familiar voice processing – as opposed to a universal vocal identity space coding for different speaker identities (Baumann & Belin, 2010; Latinus & Belin, 2011). Such person-specific voice spaces could then be projected onto universal voice spaces that code for between-speaker variability, where large areas of overlap between different person-specific voice spaces can be expected. Within such a model, researchers must then ask how listeners can access the relevant person-specific reference pattern for a familiar voice from overlapping person-specific “voice spaces.”

## Conclusion

The human voice is a rich and complex source of information to the listener. Here, we have argued for the critical importance of within-person variability as a key factor that must be addressed in any account of how, and to what extent, we can successfully extract information about person identity from the voice. We argue that approaches that emphasize between-person variability are insufficient, and we report on a growing body of work demonstrating the significant influence of within-person variability on the successful perception of identity from human vocal signals. Going forward, it will be essential to quantify and describe within-person variability in the voice and to define its relationship to between-speaker differences, for different types of natural vocal behaviors. In studies of voice-identity perception, we must account for how within-person variability challenges identity processing, but also investigate how it might assist related processes, for example the formation of person representations based on the voice. Finally, it is of interest to consider whether our observations related to voice-identity processing are grounded in acoustic perceptual processes, or whether they are more broadly reflective of amodal cognitive processes in the learning and formation of person identity “categories,” whether from the voice, face, or body.

## Footnotes

1 These findings are closely associated with the Lombard effect – while listeners can volitionally increase their vocal effort, the Lombard effect describes the involuntarily increase in vocal effort in noisy environments (Lane & Tranel, 1971).

2 Similar proposals can also be found in the speech perception literature, where variability in phoneme production is considered to be an informative signal rather than noise (for a review see Weatherholtz & Jaeger, 2015).
